# A comparative analysis of the clinical and genomic characteristics of Panton–Valentine leukocidin–positive methicillin-resistant *Staphylococcus aureus* in Korea and Japan

**DOI:** 10.1128/spectrum.00589-26

**Published:** 2026-07-21

**Authors:** Hiroshi Kaneko, Eu Suk Kim, Shiho Yokomori, Wan Beom Park, Jongtak Jung, Song Mi Moon, Kyoung-Ho Song, Jeong Su Park, Taek Soo Kim, Yuji Hirai, Hiroshi Maruyama, Mitsuhiro Kamimura, Hiroyuki Miura, Kayoko Tsuchiya, Hong Bin Kim, Hidemasa Nakaminami

**Affiliations:** 1 Department of Clinical Microbiology, School of Pharmacy, Tokyo University of Pharmacy and Life Sciences 609118, , Hachioji, Tokyo, Japan; 2 Division of Infectious Diseases, Department of Internal Medicine, Seoul National University Bundang Hospital 541267, , Seongnam, Republic of Korea; 3 Department of Internal Medicine, Seoul National University College of Medicine 198707, Seoul, Republic of Korea; 4 Division of Infectious Diseases, Department of Internal Medicine, Soonchunhyang University Seoul Hospital, and Soonchunhyang University College of Medicine 198707, Seoul, Republic of Korea; 5 Department of Laboratory Medicine, Seoul National University College of Medicine, Seoul, Republic of Korea; 6 Department of Infectious Diseases, Tokyo Medical University Hachioji Medical Center 89440, Hachioji, Tokyo, Japan; 7 Department of Surgery, Nippon Medical School Tama Nagayama Hospital 26368, Tama, Tokyo, Japan; 8 Department of Respiratory Medicine, National Hospital Organization Disaster Medical Center 13504, Tachikawa, Tokyo, Japan; 9 Department of Thoracic Surgery, Akiru Municipal Medical Centre 74143, Akiruno, Tokyo, Japan; 10 Department of Respiratory Medicine, Tachikawa Sogo Hospital 215660, Tachikawa, Tokyo, Japan; University of Calgary, Calgary, Alberta, Canada

**Keywords:** methicillin-resistant *Staphylococcus aureus*, Panton–Valentine leukocidin, USA300, ΨUSA300, ST22-PT

## Abstract

Panton**–**Valentine leukocidin (PVL) is a leukocyte-lytic toxin produced by *Staphylococcus aureus*, which is primarily associated with skin and soft tissue infections. Notably, there has been an increase in the number of cases caused by the USA300 lineage in Japan. However, the reported prevalence of USA300 is limited in other Asian countries, including Korea. This study investigated the prevalence of PVL-positive methicillin-resistant *S. aureus* (MRSA) in Korea and Japan and compared their molecular epidemiological characteristics. A total of 463 MRSA strains were analyzed, comprising 283 strains from patients visiting two hospitals in Seongnam and Seoul, Korea, and 180 strains from six hospitals in Tokyo, Japan, between 2018 and 2019. The PVL-encoding genes *lukS/F-PV* were detected using PCR. Molecular epidemiological and phylogenetic analyses were performed using next-generation sequencing. Overall, 27 (9.5%) PVL-positive MRSA strains were detected among strains isolated from Korea, and 16 (8.9%) were detected among those isolated from Japan. Genotyping of PVL-positive strains revealed that 85.2% of Korean and 81.3% of Japanese strains belonged to USA300. Most USA300 strains from Japan and Korea formed distinct clusters in phylogenetic analyses. Meanwhile, ΨUSA300 and ST22-PT, clones that are prevalent in Japan, were isolated in Korea. This study showed that USA300 strains, which are becoming more prevalent in Japan, are also present in Korea. Furthermore, this study suggests that ΨUSA300 and ST22-PT may be spreading between these two countries. Therefore, it is necessary to continue monitoring the epidemiological trends of PVL-positive MRSA clones both domestically and internationally.

**IMPORTANCE:**

Panton–Valentine leukocidin (PVL) is a major toxin produced by *Staphylococcus aureus*. Although a rapid increase in PVL-positive strains has been reported in Japan, data on PVL-positive strains in Korea remain limited. In this study, we performed a comparative analysis of PVL-positive *S. aureus* isolates from Korea and Japan. The results showed that the clinical backgrounds and genetic profiles of PVL-positive strains isolated in Korea and Japan were highly similar. Furthermore, we confirmed for the first time that clones circulating in Japan, including ΨUSA300 and ST22-PT, were also isolated in Korea. These findings provide valuable insights into the epidemiological status of PVL-positive *S. aureus* in East Asia.

## INTRODUCTION

*Staphylococcus aureus* is a commensal bacterium of humans; however, it is also a pathogen associated with various diseases, ranging from skin and soft tissue infections to more serious conditions, such as necrotizing pneumonia and endocarditis (1, 2). In particular, infections caused by methicillin-resistant *S. aureus* (MRSA), which are resistant to several antimicrobial agents, are often difficult to treat. MRSA possesses the methicillin resistance gene *mecA*, which encodes penicillin-binding protein 2′ (PBP2′) that has a low affinity for β-lactam antimicrobial agents. *mecA* is located on the staphylococcal cassette chromosome *mec* (SCC*mec*), which is a mobile genetic element on the chromosome (3). This bacterium produces several pathogenic factors, including Panton**–**Valentine leukocidin (PVL), toxic shock syndrome toxin-1 (TSST-1), staphylococcal enterotoxin (SE), and exfoliative toxin (ET). PVL is a pore-forming cytotoxin primarily associated with severe skin and soft tissue infections (4). PVL comprises proteins encoded by the *lukS-PV* and *lukF-PV* genes, with LukS-PV binding to the C5a receptor on the surface of neutrophils, followed by LukF-PV binding to exert its toxicity (5–7).

Recently, the number of highly pathogenic PVL-positive MRSA strains has increased in communities in Japan. There has been a significant increase in USA300 strains, which are prevalent in the United States (8–10). Notably, a USA300 variant, known as ΨUSA300, has spread widely (11, 12). USA300 is described as a highly virulent MRSA clone that carries type IV SCC*mec* and the arginine catabolic mobile element (ACME), which promotes skin colonization, and is classified as clonal complex (CC) 8 by multilocus sequence typing (MLST) (8, 13).

USA300 is widely prevalent in Japan and has been classified as an epidemic in Taiwan (14). On the other hand, only limited spread has been reported in other Asian countries, including Korea (15). However, it is possible that the increasing number of USA300 strains in Japan has led to its spread to neighboring Asian countries. Furthermore, a new clone, ST22-PT, has been spreading in Japan in recent years (16). This strain produces both PVL and TSST-1. Similar strains have been isolated from several other countries (17). However, its prevalence in neighboring countries remains unknown. To address this issue, we investigated the molecular epidemiological characteristics of PVL-positive MRSA isolates from Korea and Japan.

## MATERIALS AND METHODS

### Patients and bacterial strains

Non-duplicate MRSA clinical isolates were collected and stored over a 3-month period from November 2018 to January 2019 in two tertiary care teaching hospitals in Korea and six tertiary care hospitals in Japan (Table 1). In this study, all collected specimens were included, irrespective of whether they were obtained from sterile or non-sterile sites. When multiple MRSA isolates were recovered from the same patient, only one isolate was included in the analysis. In such cases, priority was given to the MRSA isolate recovered from the primary site of infection. All subsequent analyses described below were performed using MRSA clinical isolates for which all clinical data required for comparison in this study were available and which had been appropriately stored as viable strains. These MRSA isolates were screened for the presence of the *lukS-PV* and *lukF-PV* genes, which encode PVL, using polymerase chain reaction (PCR), as previously described (18). These PCR analyses were performed in the central laboratories of Korea and Japan. PVL-positive MRSA strains were sent to the Tokyo University of Pharmacy and Life Sciences for storage at −80°C and subsequent genomic analysis. Demographic, clinical, and epidemiological data were retrospectively collected from medical records maintained by infectious disease physicians or trained research nurses in each hospital using a standardized protocol. Information on the basic characteristics of the participating hospitals was also collected.

**TABLE 1:** Basic characteristics of eight participating hospitals in Korea and Japan and number of non-duplicate *Staphylococcus aureus* clinical isolates and Panton–Valentine leukocidin (PVL)-positive methicillin-resistant *S. aureus* (MRSA) in each hospital

Hospital	Country, location	Number of hospital beds	Referral class	University affiliation	Total number of *S. aureus* isolates	Number of MRSA isolates	Number of MRSA isolates ≤48 h after hospitalization	Number of screened MRSA isolates	Number of PVL-positive MRSA isolates
A	Korea, Seoul	>1,500	Tertiary	Yes	452	220	103	146	9
B	Korea, Seongnam	1,000–1,500	Tertiary	Yes	418	180	74	137	18
C	Japan, Tokyo	300–500	Tertiary	No	93	28	9	25	2
D	Japan, Tokyo	300–500	Tertiary	No	172	41	4	25	4
E	Japan, Tokyo	300–500	Tertiary	Yes	144	73	5	49	2
F	Japan, Tokyo	300–500	Tertiary	No	138	38	2	7	0
G	Japan, Tokyo	500–1,000	Tertiary	Yes	226	83	15	37	3
H	Japan, Tokyo	<300	Tertiary	No	117	48	24	37	5
Sum					1,760	711	236	463	43

### Definitions

Infection was classified as community-onset (CO) or hospital-onset (HO) according to when the MRSA-positive specimen was obtained (within or after 48 h of hospitalization, respectively). Community-associated (CA) infection was defined as the absence of any of the recognized healthcare-associated (HA) risk factors for MRSA acquisition or infection, as described previously (2). Sterile clinical specimens included blood, deep tissue abscesses, cerebrospinal fluid, ascites, pleural fluid, pericardial fluid, joint fluid, and bone tissue samples. Non-sterile clinical specimens included pus, sputum, urine, and skin swabs. The primary site of infection was determined according to the best practices of the infectious disease physician at each hospital.

### Genomic analysis

The genomic DNA from the USA300 representative strains—A776, N1404, THI2019-8, TP1611, TP1617, and TSI972—was extracted using a DNeasy Blood & Tissue Kit (Qiagen, Hilden, Germany). Whole-genome sequencing of these strains was performed using the DNBSEQ-G400 (MGI Tech Co., Ltd., Beijing, China) with 200-bp paired-end reads and GridION (Oxford Nanopore Technologies Ltd., Oxford, UK), as previously described (19). Genomic DNA was extracted from the remaining PVL-positive MRSA strains after treatment with lysostaphin (FUJIFILM Wako Pure Chemical Corporation, Osaka, Japan), proteinase K (FUJIFILM Wako), and AMPure XP (Beckman Coulter, Inc., CA, USA). The library was prepared using a KAPA HyperPlus Kit (Roche Diagnostics K.K., Tokyo, Japan). Genome data were obtained using the NextSeq 2000 (Illumina, Inc., CA, USA) with 150-bp paired-end reads. The complete and draft genome sequences were deposited in GenBank and the Sequence Read Archive, respectively (Supplemental Material). CCs were determined from the obtained genome data by querying the PubMLST database (https://pubmlst.org/). Using ResFinder (20) and VirulenceFinder (21), resistance genes and virulence factors, respectively, were detected with thresholds of identity ≥80%, coverage ≥60%, and depth ≥10. SCC*mec* types were determined using SCC*mec*Finder (22). Based on the analysis results, CC8 strains harboring SCC*mec* type IV with a 12-bp deletion in the *ccrB2* gene (type ΨIV) were classified as ΨUSA300, whereas CC22 strains harboring TSST-1 were classified as ST22-PT. Single nucleotide polymorphism (SNP) calling was performed using Snippy (https://github.com/tseemann/snippy) by mapping draft genome reads to FPR3757 (13) for CC8 strains and TPS5614 (16) for CC22 strains, with depth ≥10, proportion ≥80%, and quality ≥60. A maximum-likelihood phylogenetic tree based on SNPs in the core genome was generated using PhaME (23) and RAxML (24), and visualized using FigTree v1.4.4 (http://tree.bio.ed.ac.uk/software/figtree/). Based on previous reports (12, 14–17, 25), genomic data from strains exhibiting characteristics similar to those isolated in the present study were used as comparative references (Supplemental Material). For the phylogenetic analysis of CC8 isolates, we used genome data from all isolates classified as USA300 North American epidemic (USA300-NAE) in the report by Strauss et al. (25), as well as selected genome data from isolates previously recovered in East Asian countries, including Taiwan, South Korea, and Japan (12, 14, 15). For the phylogenetic analysis of CC22 isolates, we used genome data from all isolates classified as ST22-PT or closely related clones in the reports by Zhou et al. and Kaneko et al. (16, 17).

### Antimicrobial susceptibility testing

The minimum inhibitory concentrations (MICs) of the antimicrobial agents were determined using the agar dilution method according to the Clinical and Laboratory Standards Institute (CLSI) guidelines (M07-A11) (26). The following antimicrobial agents were used: ampicillin (Sigma-Aldrich Co., LLC, MO, USA), oxacillin (Tokyo Chemical Industry Co., Ltd., Tokyo, Japan), gentamicin (FUJIFILM Wako), clarithromycin (Tokyo Chemical Industry), clindamycin (Tokyo Chemical Industry), levofloxacin (Tokyo Chemical Industry), minocycline (Tokyo Chemical Industry), and vancomycin (FUJIFILM Wako). The breakpoints for these antimicrobial agents were determined based on CLSI interpretation criteria (M100-S32) (27).

### Statistical analysis

Statistical analyses were performed for risk factors and resistance rates using the Mann–Whitney U test or chi-squared test (or Fisher’s exact test, depending on the type of variable) with IBM SPSS Statistics, version 25. Statistical significance was set at *P* < 0.05.

## RESULTS

### Prevalence of PVL-positive MRSA in Korea and Japan

A total of 1,760 non-duplicate *S. aureus* strains were isolated from eight hospitals during the study. Of these, 711 were MRSA, including 236 CO-MRSA isolates that were obtained from patients within 48 h of hospitalization. Screening for the *lukS/F-PV* genes was performed on 463 MRSA strains. Of these, 9.3% (43 strains) were PVL-positive (Table 1). PVL-positive MRSA was detected at frequencies of 9.5% (27 strains) and 8.9% (16 strains) in Korea and Japan, respectively. No significant differences were observed between the two countries.

Further, there were no significant differences between the two countries in terms of the clinical characteristics of the PVL-positive MRSA infections (Table 2). Infections were more prevalent among men, and most patients were aged 65 years or older (77.8% and 62.5% in Korea and Japan, respectively). Almost half of the samples were collected in the emergency room (ER) or outpatient department (OPD), and 22.2% (6 strains) and 50% (8 strains), respectively, were sterile clinical specimens. The most common primary sites of PVL-positive MRSA infection were skin and soft tissue infections (SSTIs) (33.3% and 56.3% in Korea and Japan, respectively), followed by pneumonia (25.9% and 25.0%, respectively). Epidemiologically, PVL-positive MRSA was more commonly classified as CA-MRSA in Japan (50.0%) than in Korea (25.9%), although this difference was not statistically significant.

**TABLE 2:** Comparison of clinical and molecular characteristics of Panton–Valentine leukocidin (PVL)-positive methicillin-resistant *Staphylococcus aureus* (MRSA) in Korea and Japan^*a*^

	PVL-positive MRSA infections in Korea (*n* = 27)	PVL-positive MRSA infections in Japan (*n* = 16)	*P* value
Gender			0.534
Male	16 (59.3%)	11 (68.8%)	
Female	11 (40.7%)	5 (31.3%)	
Age mean ± SD (years)	67.6 ± 22.3	65.3 ± 26.2	0.753
Age median (IQR) (years)	75 (66–82)	71 (56–83)	0.318
Age group			0.558
<10	1 (3.7%)	1 (6.3%)	
10–64	5 (18.5%)	5 (31.3%)	
≥65	21 (77.8%)	10 (62.5%)	
Specimen collection site			0.168
ER or OPD	11 (40.7%)	10 (62.5%)	
GW or ICU	16 (59.3%)	6 (37.5%)	
Specimen sterility			0.093
Sterile	6 (22.2%)	8 (50%)	
Non-sterile	21 (77.8%)	8 (50%)	
Primary site of infection			0.101
SSTIs	9 (33.3%)	9 (56.3%)	
Pneumonia	7 (25.9%)	4 (25.0%)	
Others^*b*^	11 (40.7%)	3 (18.8%)	
Epidemiological characteristic			0.263
Community-onset			
Community-associated	7 (25.9%)	8 (50.0%)	
Healthcare-associated	10 (37.0%)	3 (18.8%)	
Hospital-onset	10 (37.0%)	5 (31.3%)	

^*a*^ SD, standard deviation; IQR, interquartile range; ER, emergency room; OPD, outpatient department; GW, general ward; ICU, intensive care uint; SSTIs, skin and soft tissue infections.

^*b*^ Others included bone and joint infection, otitis media, endovascular infection, intraabdominal infection, urinary tract infection, primary bacteremia, and simple colonization.

### Basic molecular epidemiological features of PVL-positive MRSA in Korea and Japan

SCC*mec* typing was performed to identify the origin of the PVL-positive MRSA isolates. SCC*mec* type IV was present in 15 Japanese strains (93.8%), whereas type V was present in one strain (6.3%). However, none of the strains isolated in this study carried SCC*mec* type ΨIV, which has been reported to be prevalent in Japan. Among the Korean strains, type IV was present in 25 strains (92.6%) and type V in one strain (3.7%). SCC*mec* types I–III were not detected in any of the Korean or Japanese strains. Of note, SCC*mec* type ΨIV was detected in one Korean isolate (3.7%). Therefore, the most commonly detected SCC*mec* type in both Korean and Japanese isolates was type IV, which is prevalent in MRSA isolates from community settings.

**Fig 1:**
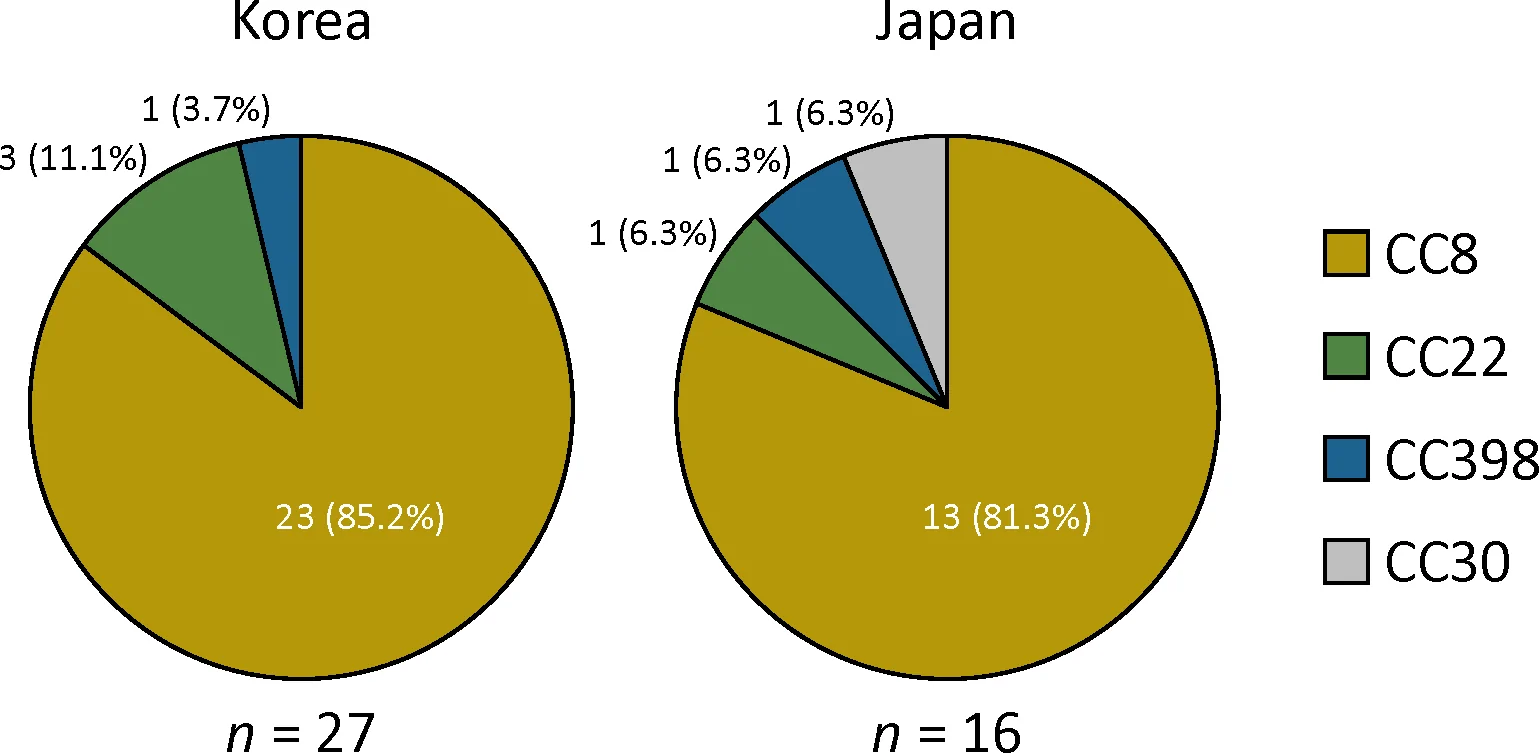
Genotypes of Panton–Valentine leukocidin (PVL)-positive methicillin-resistant *Staphylococcus aureus* (MRSA) isolates from Korea and Japan.

To clarify the phylogenetic relationship between the strains isolated from the two countries, MLST was performed to determine the genotypes of PVL-positive MRSA (Fig. 1). Overall, 85.2% (23 strains) of the Korean strains and 81.3% (13 strains) of the Japanese strains were classified as CC8, which is represented by USA300. Most CC8 strains harbored ACME type I. Furthermore, three strains from Korea and one strain from Japan belonged to CC22. One strain each from Korea and Japan belonged to CC398. Only one strain from Japan belonged to CC30.

### Comparative genomic analysis of PVL-positive MRSA in Korea and Japan

**Fig 2:**
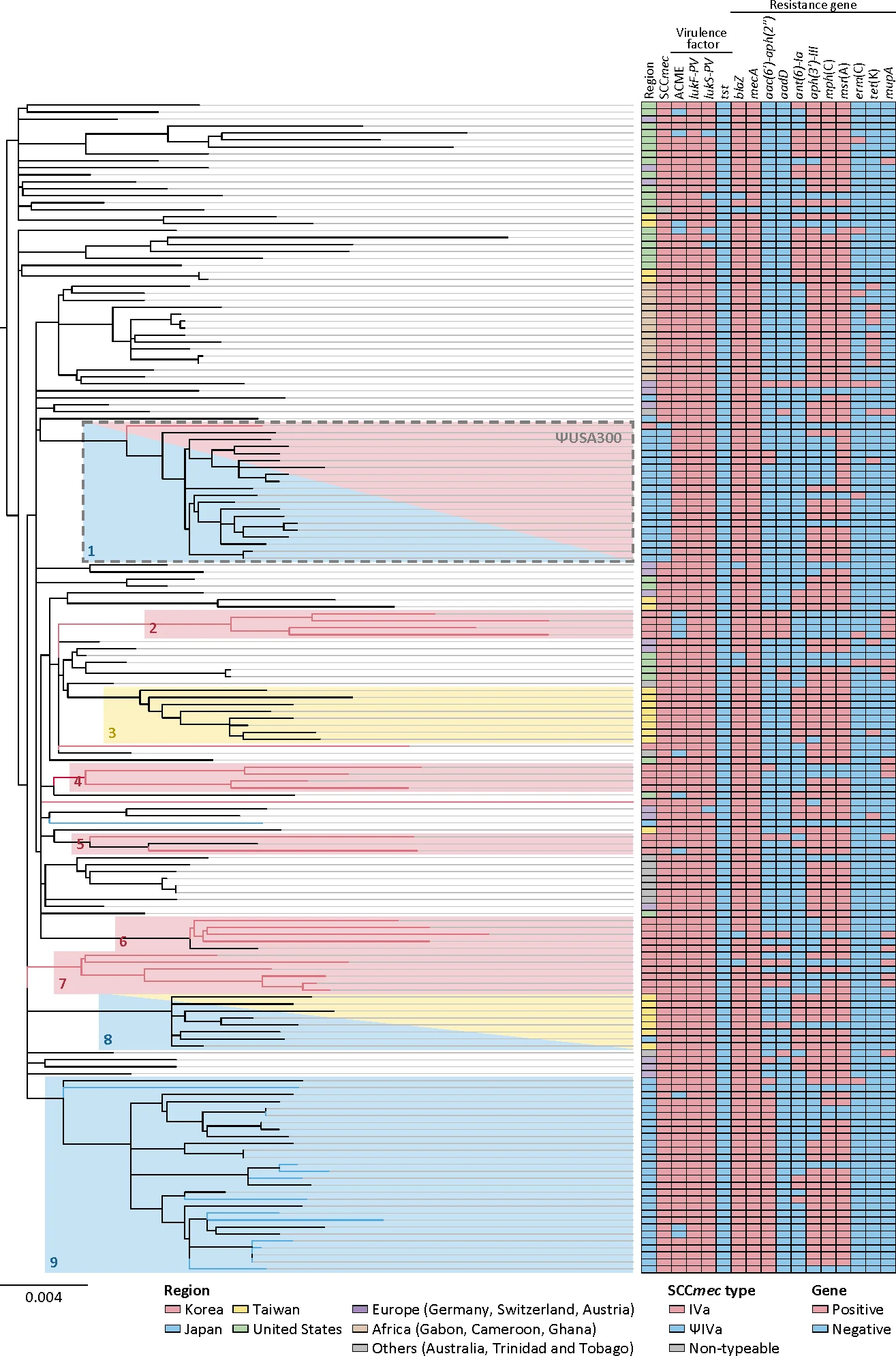
Comparative genomic analysis of CC8 *Staphylococcus aureus* isolates. Isolates collected in this study are highlighted by red (Korea) and blue (Japan) lines. The scale bar indicates the average number of substitutions per base position. A total of 4,819 SNPs within the core genome were compared.

**Fig 3:**
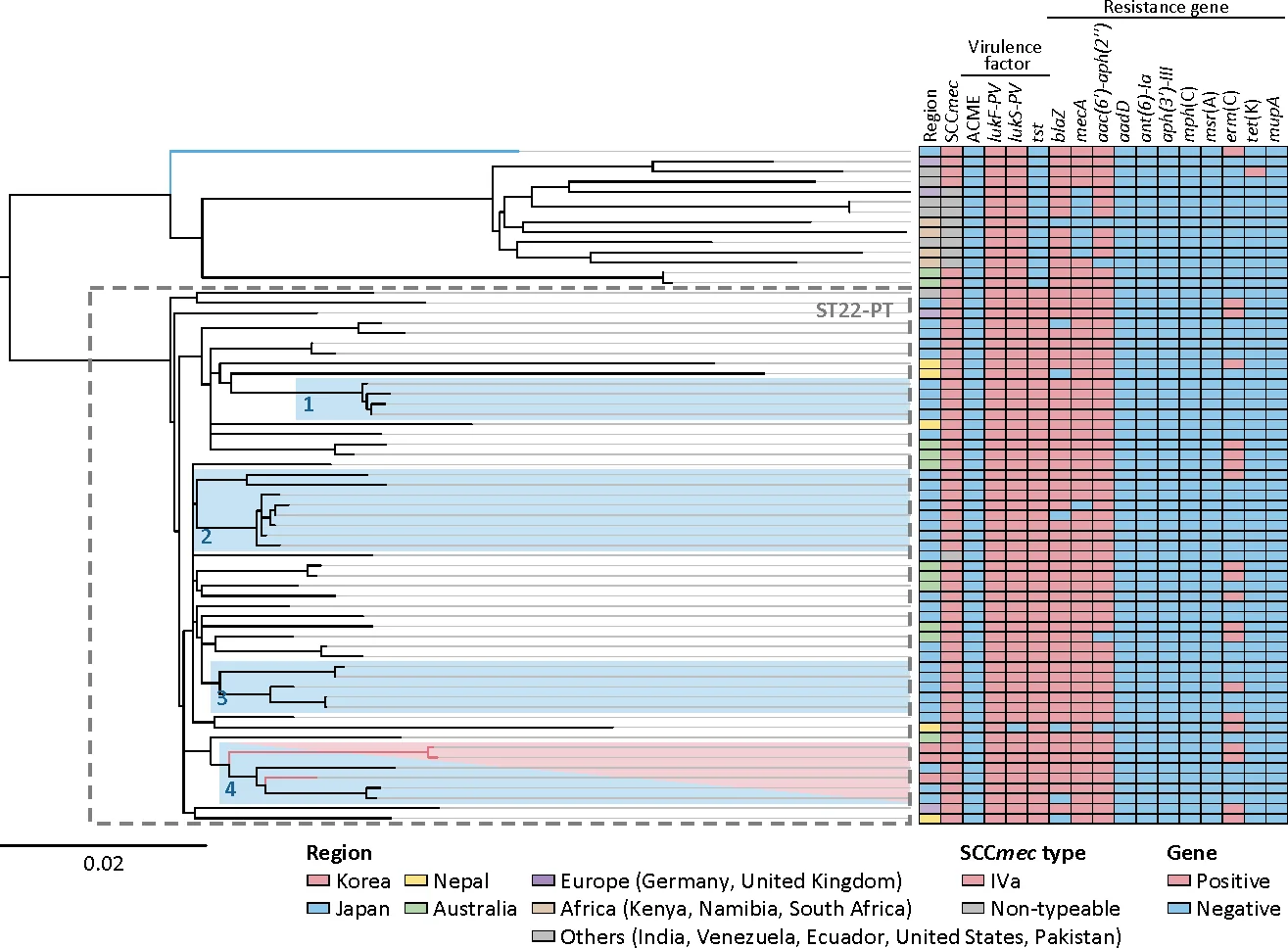
Comparative genomic analysis of CC22 *Staphylococcus aureus* isolates. Isolates collected in this study are highlighted by red (Korea) and blue (Japan) lines. The scale bar indicates the average number of substitutions per base position. A total of 2,262 SNPs within the core genome were compared.

To enable more detailed comparisons, a comparative genomic analysis was performed for each genotype. Several strains isolated from various regions worldwide were included for comparison. All the PVL-positive CC8 strains isolated in this study belonged to the USA300-NAE cluster (25). However, they formed several subclusters (Fig. 2; Table S1). Most strains isolated in Japan belonged to clusters no. 1 (ΨUSA300) and no. 9, whereas those isolated in Korea were distributed across several clusters. Among the strains included in the phylogenetic analysis, the Japanese strains isolated in this study were predominantly concentrated in cluster no. 9. The ΨUSA300 strain isolated from Korea was assigned to cluster no. 1, together with the strains isolated in Japan. Additionally, although these strains were not included among the isolates examined in this study, some strains isolated from Taiwan and Japan were assigned to cluster no. 8, which represented a mixed cluster. A comparison of antimicrobial resistance gene profiles revealed that all but one strain in cluster no. 9 harbored *aac(6′)-aph(2*″), which confers resistance to aminoglycosides. In contrast, the presence of other major resistance genes varied among the strains. None of the strains harbored *tst*, the gene encoding TSST-1. Phylogenetic analysis of CC22 strains showed that ST22-PT strains, which carried *tst*, formed a cluster distinct from that of *tst*-negative strains (Fig. 3; Table S2). The ST22-PT strains were further divided into several subclusters. Of the four PVL-positive CC22 strains isolated in this study, three were from Korea and were assigned to cluster no. 4, together with the previously reported ST22-PT strains. A comparison of antimicrobial resistance gene profiles revealed that *aac(6′)-aph(2*″) was present in most of the analyzed CC22 strains, whereas other major resistance genes were detected at generally low frequencies. Detailed analysis of the PVL-positive CC398 strain was not performed as the authors have previously reported this strain (19).

### Antimicrobial susceptibility of PVL-positive MRSA in Korea and Japan

We compared the antimicrobial susceptibilities of CC8 strains (Table 3). A significant difference in gentamicin-resistance rates was observed between the CC8 strains isolated from Japan and those isolated from Korea (*P* < 0.01). This result also correlated with the tendency of the strains to harbor aminoglycoside resistance genes (Fig. 2). Additionally, a certain degree of variation was noted in the distribution of levofloxacin MIC values, although the differences were not significant. Conversely, the susceptibility of USA300 strains isolated from both countries to other antimicrobial agents was almost identical. All the strains were susceptible to anti-MRSA drugs and minocycline.

**TABLE 3:** Antimicrobial susceptibility of Panton–Valentine leukocidin (PVL)-positive clonal complex (CC) 8 methicillin-resistant *Staphylococcus aureus* in Korea and Japan^*a*^

Antimicrobial agent	Korea (*n* = 23)			Japan (*n* = 13)		
	Range	MIC_50_/MIC_90_	Number of resistant strains (%)	Range	MIC_50_/MIC_90_	Number of resistant strains (%)
Ampicillin	1 to 32	16/32	23 (100.0)	8 to 32	16/32	13 (100.0)
Oxacillin	8 to 64	32/64	23 (100.0)	8 to 64	32/64	13 (100.0)
Gentamicin	0.13 to 64	0.13/16	4 (17.4)	0.13 to 128	64/64	11 (84.6)^*b*^
Arbekacin	0.13 to 1	0.13/0.5	–	≤0.06 to 1	0.25/0.5	–
Clarithromycin	≤0.06 to 128	32/128	14 (60.9)	≤0.06 to 64	32/32	8 (61.5)
Clindamycin	≤0.06 to 128	≤0.06/0.13	1 (4.3)	≤0.06 to ≤0.06	≤0.06/≤0.06	0 (0.0)
Levofloxacin	4 to 128	8/32	23 (100.0)	4 to 16	4/8	13 (100.0)
Minocycline	≤0.06 to ≤0.06	≤0.06/≤0.06	0 (0.0)	≤0.06 to 0.13	≤0.06/0.13	0 (0.0)
Vancomycin	1 to 1	1/1	0 (0.0)	1 to 1	1/1	0 (0.0)

^*a*^ MIC_50_/MIC_90_, MIC required to inhibit the growth of 50%/90% of the strains, respectively; –, breakpoint has not been defined.

^*b*^ *P* < 0.01 compared with the percentage of resistant strains in Korea by Fisher's exact test.

## DISCUSSION

In the present study, the isolation rate of PVL-positive MRSA was lower than that reported in the United States (28), and no significant difference was observed between Korea and Japan. The clinical characteristics of PVL-positive MRSA infections reported in the two countries were comparable. The overall clinical and epidemiological features were very similar between the two countries; however, there was a tendency toward a higher proportion of SSTIs and a higher frequency of epidemiological classification as CA-MRSA in Japan. Despite the close geographic proximity and frequent population movement between Korea and Japan, the two countries differ in their healthcare systems, patterns of antimicrobial use, and resistance profiles of major bacterial pathogens. In this context, the clinical and epidemiological similarities observed in this study suggest that the microbiological characteristics of PVL-positive MRSA may play an important role in shaping its epidemiology in both countries, potentially outweighing the differences in healthcare practices. Therefore, further investigations into the underlying microbiological and molecular characteristics of these strains are warranted.

The PVL-positive MRSA isolates obtained in this study were primarily recovered from patients with SSTIs or pneumonia. This finding is consistent with previous reports indicating an association between PVL production and the development of SSTIs and pneumonia (11, 29, 30). Although this remains controversial (31), PVL has been suggested to be associated with increased severity of these diseases (32). In addition, although the contribution of TSST-1 to pathogenicity remains unclear, severe cases caused by the newly emerging clone ST22-PT have also been reported (16, 33). Recent studies have reported an increasing prevalence of PVL-positive MRSA in community settings in Japan (11, 12), highlighting the need for continued surveillance to better understand temporal trends and transmission dynamics of these strains in the region.

Of the PVL-positive MRSA strains isolated, 85.2% of Korean strains and 81.3% of Japanese strains were CC8, indicating that USA300 is prevalent in both countries. This study also suggests that the origins of the CC8 strains isolated in Korea and Japan are different, and that USA300, which was introduced from abroad, has evolved independently in both countries. Given that previous studies have reported clonal outbreaks specific to institutions or geographic regions (34, 35), some of the clusters observed among CC8 isolates, such as clusters no. 6, no. 7, and no. 9, are likely to represent locally prevalent clones circulating in the regions where the participating institutions were located. In particular, the high prevalence of *aac(6′)-aph(2*″) among strains belonging to cluster no. 9 may reflect adaptive evolution. Additionally, the findings of this study indicate that ΨUSA300 strains may have spread between the two countries. This study is the first to report the isolation of ΨUSA300 outside Japan. Furthermore, ST22-PT, a strain prevalent in Japan, was also isolated in Korea. To our knowledge, this is the first report of ST22-PT isolation in Korea. The high genetic similarity between Korean and Japanese strains strongly suggests cross-border transmission, indicating that ST22-PT currently spreading in Japan has been introduced into Korea.

Antimicrobial susceptibilities of CC8 strains isolated from the two countries exhibited similarities. In addition to anti-MRSA drugs, minocycline has been identified as an effective antimicrobial agent against PVL-positive MRSA strains circulating in Korea and Japan. The gentamicin resistance rate was significantly different between the two countries—17.4% in Korean strains and 84.6% in Japanese strains. This is believed to be due to the acquisition and loss of resistance genes through horizontal transmission via plasmids and transposons.

A limitation of this study is that the analyzed isolates were obtained from a limited number of institutions and, therefore, did not represent a comprehensive nationwide collection. In general, the epidemiological characteristics of *S. aureus* can vary substantially by region, even within a single country. Therefore, the present findings may not fully reflect the overall epidemiological trends in Korea and Japan. Nevertheless, the observation that PVL-positive MRSA isolates recovered over the same period from both countries exhibited similar characteristics—based on the analysis of a relatively large number of isolates—represents an important finding considering the epidemiological relatedness between the two countries.

MRSA spreads primarily through direct contact with colonized or infected individuals. With increasing globalization and movement of people within Asian countries, continued adherence to standard infection-prevention practices remains relevant in both healthcare-associated and community settings given the circulation of PVL-positive MRSA in the community. As globalization continues to increase, it is essential to monitor trends in human movement and MRSA prevalence. In addition, the prevalence of USA300 and ST22-PT strains in Japan is increasing (11, 16). Because many individuals in hospitals are susceptible to infection, stricter infection control measures are necessary. It is crucial to monitor the prevalence of PVL-positive MRSA clones both domestically and internationally and to identify the most appropriate antimicrobial agents for effective treatment.

## Data Availability

All data generated or analyzed during this study are included in this article. No additional data have been deposited in a public repository. The authors certify that they will comply with the journal’s data sharing policy.

## AFTER EPUB

*[This article was published on 21 July 2026 with an error in the affiliations. The affiliations were corrected in the current version, posted on 18 August 2026.]*

## SUPPLEMENTAL MATERIAL

Supplemental Material spectrum.00589-26-s0001.xlsx Detailed information on the bacterial strains used in the comparative genomic analysis.

Table S1 spectrum.00589-26-s0002.xlsx Number of SNPs among CC8 *Staphylococcus aureus* isolates.

Table S2 spectrum.00589-26-s0003.xlsx Number of SNPs among CC22 *Staphylococcus aureus* isolates.

ASM does not own the copyrights to Supplemental Material that may be linked to, or accessed through, an article. The authors have granted ASM a non-exclusive, world-wide license to publish the Supplemental Material files. Please contact the corresponding author directly for reuse.
